# Folic Acid and Vitamins D and B12 Correlate With Homocysteine in Chinese Patients With Type-2 Diabetes Mellitus, Hypertension, or Cardiovascular Disease

**DOI:** 10.1097/MD.0000000000002652

**Published:** 2016-02-12

**Authors:** Xudong Mao, Xubin Xing, Rong Xu, Qing Gong, Yue He, Shuijun Li, Hongfu Wang, Cong Liu, Xin Ding, Rishu Na, Zhiwen Liu, Yi Qu

**Affiliations:** From the Department of Geriatrics (XM, YQ, RX, QG, YH); Central Laboratory (SL); Department of Endocrinology, Shanghai Xuhui Central Hospital, Shanghai Clinical Center, Chinese Academy of Sciences (CL, XD, ZL, RN); Department of Cardiology, Ruijin Hospital Luwan Branch, Shanghai Jiaotong University School of Medicine, (XX); and Institute of Radiation Medicine, Shanghai Medical College, Fudan University, Shanghai, China (HW).

## Abstract

Elevated serum homocysteine has been shown to be a risk factor for hypertension, cardiovascular disease (CVD), and type-2 diabetes mellitus (T2DM).

We characterized the relationships between the serum levels of homocysteine, folic acid, and vitamins D2, D3, and B12 in patients with T2DM, CVD, and hypertension in Shanghai, China. The levels of these serum biochemical markers were determined for 9311 Chinese patients (mean age: 79.50 ± 13.26 years) with T2DM (*N* = 839), hypertension (*N* = 490), or CVD (*N* = 7925). The demographic and serum biochemical data were compared using an analysis of variance. We performed stratified analyses using Pearson linear regression to investigate correlations between the different variables in the T2DM, CVD, and hypertension groups and in patients aged < 50, 50 to 64, 65 to 80, and ≥80 years. A subgroup analysis was also performed to identify correlations between the serum biochemical markers. Stratified chi-squared analyses were performed based on the levels of folic acid and total vitamin D.

In all 3 patient groups, elevated levels of vitamin D2 and homocysteine were observed, whereas the levels of folic acid and vitamins D3 and B12 were lower than the reference range for each serum marker (*P* < 0.05 for all). The linear regression and stratified analyses showed that the highest levels of folic acid and vitamins D2 and D3 correlated with the lowest level of homocysteine in T2DM, CVD, and hypertension patients (*P* < 0.05 for all), whereas the highest level of vitamin B12 correlated with a lowest level of homocysteine in CVD patients only (*P* < 0.05).

Our results indicate that the contributions of both vitamin D2 and vitamin D3 should be considered in investigations of the effects of vitamin D supplements in T2DM, CVD, and hypertension patients. Our findings warrant future studies of the benefits of vitamin D and folic acid supplements for reducing the risk of T2DM, CVD, and hypertension in elderly Chinese people, as well as the benefits of vitamin B12 supplements for reducing the risk of CVD alone.

## INTRODUCTION

Socioeconomic improvements have been accompanied by changes in nutritional intake that are associated with increases in the incidences of type-2 diabetes mellitus (T2DM), hypertension, and cardiovascular disease (CVD) in developing regions worldwide.^1^ Various strategies have been proposed for the prevention of these diseases, including lifestyle modifications and pharmacological interventions. Recently, there has been a growing interest in investigating possible associations between these diseases and the intake of certain micronutrients, including D and B vitamins.^1–3^

The D vitamins are a group of fat-soluble compounds that includes vitamin D2 (ergocalciferol) and vitamin D3 (cholecalciferol). The conversion of 7-dehydrocholesterol to vitamin D3 in the skin serves as the primary natural source of vitamin D in humans.^4^ Vitamins D2 and D3 are naturally present in relatively few foods, but both compounds are used as supplements in various food items, such as milk products. Vitamins D2 and D3 are converted to their biologically active forms by hydroxylation in the liver to 25-hydroxyvitamin D2 and 25-hydroxyvitamin D3, respectively. Vitamin D functions in a variety of physiological processes, including calcium homeostasis, bone metabolism, and neuroimmunomodulation.^5^ Epidemiological studies have suggested that vitamin D deficiency is associated with T2DM,^3,6^ CVD,^7^ and hypertension.^8^

Vitamins B9 (folic acid) and B12 (cobalamin) are essential, water-soluble micronutrients. Folic acid is required for normal cell division and growth through its roles in DNA synthesis, repair, and methylation and amino acid metabolism. Vitamin B12 is also involved in DNA synthesis, as well as fatty acid and amino acid metabolism. Previous studies have suggested that vitamin B12 and folic acid are associated with the development of adverse serum lipid profiles and stroke in patients with T2DM^9^ and hypertension,^10^ respectively. Deficiencies in both vitamins cause elevated serum homocysteine^11^ by inhibiting its conversion to methionine, and elevated serum homocysteine has been shown to be a risk factor for reduced cognitive function,^12^ hypertension,^13^ CVD,^13–15^ T2DM,^16,17^, and T2DM-related complications.^18,19^

The majority of studies of the relationships between homocysteine, vitamin D, and B vitamins and the incidence of T2DM, CVD, and hypertension have been conducted in Europe and North America, and relatively few studies have investigated the potential risks associated with these serum biochemical markers in Asian people. Therefore, it remains largely unclear as to whether vitamin B12, folic acid, and vitamin D are associated with the development of T2DM, hypertension, or CVD among people in Asian countries, such as China, in which substantial dietary changes have occurred in recent decades. In our present study, we examined the relationships between the serum levels of folic acid, homocysteine, and vitamins D2, D3, and B12 in patients with T2DM, hypertension, or CVD in Shanghai, China, the most populous Chinese metropolitan area.^20^

## PARTICIPANTS AND METHODS

### Participants

We reviewed 9311 patients with T2DM, hypertension, or CVD for inclusion in our study who received outpatient treatment in the Endocrinology Department of Shanghai Xuhui District Central Hospital between January 2012 and June 2014. The diagnoses were based on the relevant diagnostic criteria in International Classification of Diseases, Ninth Revision, Clinical Modification. The CVD cases included patients with coronary heart disease, cerebral infarction, cerebral embolism, lacunar cerebral infarction, vertebral basilar artery blood supply insufficiency, cerebral infarction sequelae, cerebral hemorrhage, subarachnoid hemorrhage, or cerebral hemorrhage sequelae. Patients with impaired hepatic or renal function (serum creatinine > 120 μmol/L), hyperthyroidism, or hypothyroidism and those who were receiving thyroid hormone therapy were excluded from our study. Written informed consent was obtained from all of the patients before their participation in our study. Our study protocol was approved by the Ethics Committee of Shanghai Xuhui Central Hospital and was performed in adherence to the Declaration of Helsinki with regard to ethical principles for research involving human subjects.

### Study Variables

Fasting serum levels of vitamins D2 and D3 were measured as 25-hydroxyvitamin D2 and 25-hydroxyvitamin D3, respectively, according to a previously described liquid chromatography-tandem mass spectrometry (LC-MS/MS) method^21,22^ using deuterated 25-hydroxyvitamin D2 and D3 as internal standards in an API 4000 Tandem Mass Spectrometer (AB Sciex) equipped with a Shimadzu series liquid chromatograph. The fasting level of homocysteine was also measured using an LC-MS/MS method, as described previously.^23^ The Analyst, version 1.5, software was used for data collection and quantitative analysis. The hepatic and renal serum profiles were recorded using an Advia 2400 Clinical Chemistry System (Siemens). The serum levels of folic acid and vitamin B12 were measured using an Advia Centaur XP Immunoassay System (Siemens). The accuracy of quality control for low, medium, and high concentrations was between 85% and 115%, and precision was within 15%. Our analysis of the levels of biochemical markers was based on the following reference ranges for healthy subjects ≥65 years of age, which had been established at Shanghai Xuhui District Central Hospital prior to the study period: Creatinine, 40 to 120 μmol/L; folic acid, >5.38 ng/mL; vitamin B12, 0.211 to 0.911 ng/mL; homocysteine, 5.0 to 15 μmol/L; vitamin D2, 2.42 to 22.4 ng/mL; vitamin D3, 10 to 55 ng/mL.

### Statistical Analysis

All of the statistical analyses were performed using the SPSS, version18.0, software (IBM). The continuous data are presented as the median and interquartile range. Differences between the proportions of men and women in the T2DM, CVD, and hypertension groups were evaluated using a chi-squared analysis., whereas intergroup differences in age, medication use, duration of disease, and the levels of the serum biochemicals were evaluated using the Kruskal–Wallis test. We performed stratified analyses using Pearson linear regression to investigate correlations between the different variables in the T2DM, CVD, and hypertension groups and in patients aged < 50, 50 to 64, 65 to 80, and ≥80 years, with the level of statistical significance set at *P* < 0.001. Stratified chi-squared analyses were performed based on the levels of folic acid and total vitamin D, with the level of statistical significance set at *P* < 0.05.

## RESULTS

### Participant Characteristics

Fifty-seven patients were excluded from our study, leaving 9254 patients with T2DM, CVD, or hypertension in our study cohort, which consisted 5123 men (55.36%) and 4131women (44.64%) who ranged in age from 31 to 104 years, with a mean age for our entire cohort was 79.50 ± 13.26 years. No significant intergroup difference in sex was observed. The T2DM group included 839 cases (9.07%). The hypertension group included 490 cases (5.30%), and the CVD group included 7925 cases (85.64%).

### Serum Biochemical Markers

The levels of the various serum biochemical markers in the 3 patient groups are shown in Table 1. In all 3 groups, elevated levels of homocysteine were observed, whereas the levels of folic acid were lower than the reference value. The highest levels of creatinine (*P* = 0.1819), vitamin D2 (*P* < 0.0001), and folic acid (*P* < 0.0001) were observed in the T2DM group. The highest level of vitamin D3 was observed in the CVD group (*P* < 0.0001). The levels of vitamin B12 in the T2DM and hypertension groups were higher than that in the CVD group (< 0.0001), and the levels of homocysteine in the T2DM and CVD groups were higher than that in the hypertension group (*P* = 0.0118). The lowest levels of vitamin B12, folic acid, vitamin D2, and creatinine were observed in the CVD group. The lowest levels of homocysteine and vitamin D3 were observed in the hypertension group.

**TABLE 1:**
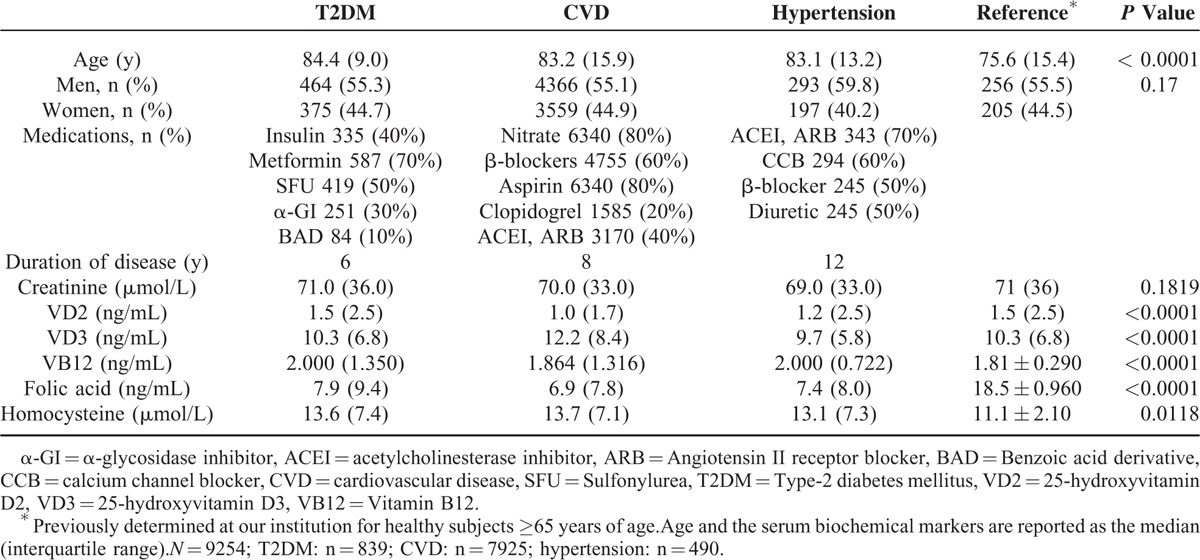
Demographic Characteristics and Levels of Serum Biochemical Markers in Patients With Type-2 Diabetes Mellitus, Hypertension, or Cardiovascular Disease

### Correlations Between Serum Biochemical Markers and Age in Different Age Groups

The results of the stratified analysis of correlations between age, homocysteine, folic acid, creatinine, vitamin D2, vitamin D3, and vitamin B12 in the various age groups are presented in Table 2. A significant correlation was observed between homocysteine and creatinine in patients < 50 years of age (*P* < 0.001). Significant correlations were observed between homocysteine and vitamin B12, homocysteine and creatinine, folic acid and vitamin D2, folic acid and vitamin D3, and folic acid and creatinine in patients 50 to 64 years of age (*P* < 0.001). Significant correlations were observed between homocysteine and Vitamin B12, homocysteine and creatinine, folic acid and vitamin D2, and folic acid and vitamin D3 in patients 65 to 84 years of age (*P* < 0.001). The significant correlations between serum biochemical markers in patients ≥80 years of age were as follows: homocysteine and vitamin B12; homocysteine and creatinine; folic acid and vitamin D2; folic acid and vitamin D3; folic acid and creatinine; folic acid and vitamin B12; homocysteine and folic acid; homocysteine and vitamin D2; homocysteine and vitamin D3; vitamin D2 and vitamin D3; and vitamin D2 and vitamin B12. Vitamin D2, vitamin B12, and creatinine each correlated with age in patients ≥80 years of age.

**TABLE 2:**
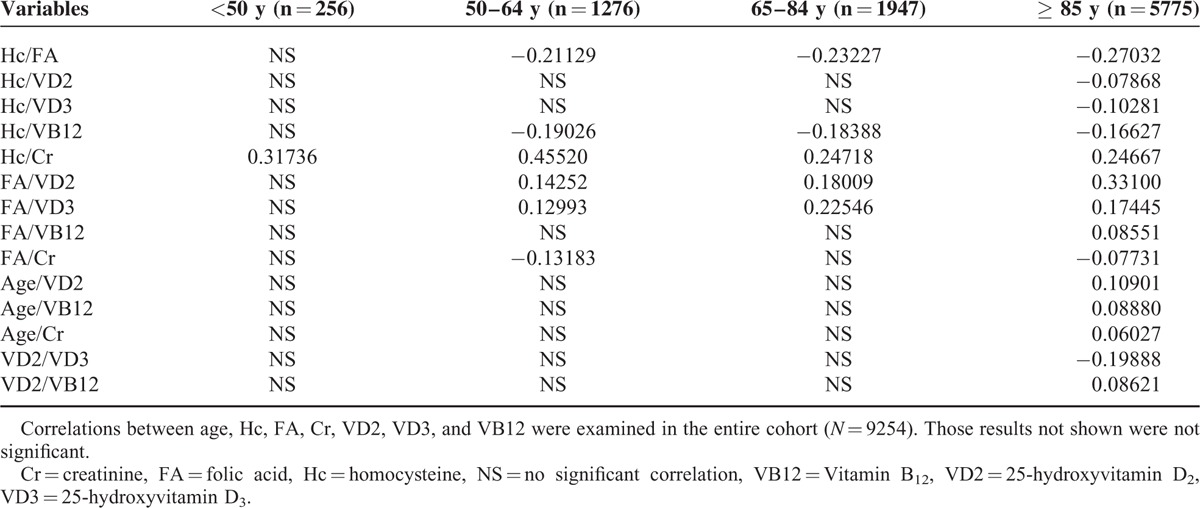
Significant Correlations (*P* < 0.001) Between Study Variables Stratified by Age

### Correlations Between Serum Biochemical Markers in T2dm Patients

Correlations between the serum biochemical markers in patients with T2DM are shown in Table 3. Vitamin D2 positively correlated with folic acid, and negatively correlated with vitamin D3 and homocysteine. Vitamin D3 positively correlated with folic acid and negatively correlated with homocysteine. Vitamin B12 negatively correlated with homocysteine. Folic acid negatively correlated with homocysteine. Increases in folic acid and vitamins D3 and B12 correlated with decreases in homocysteine and creatinine, which would tend to relieve vascular sclerosis.

**TABLE 3:**
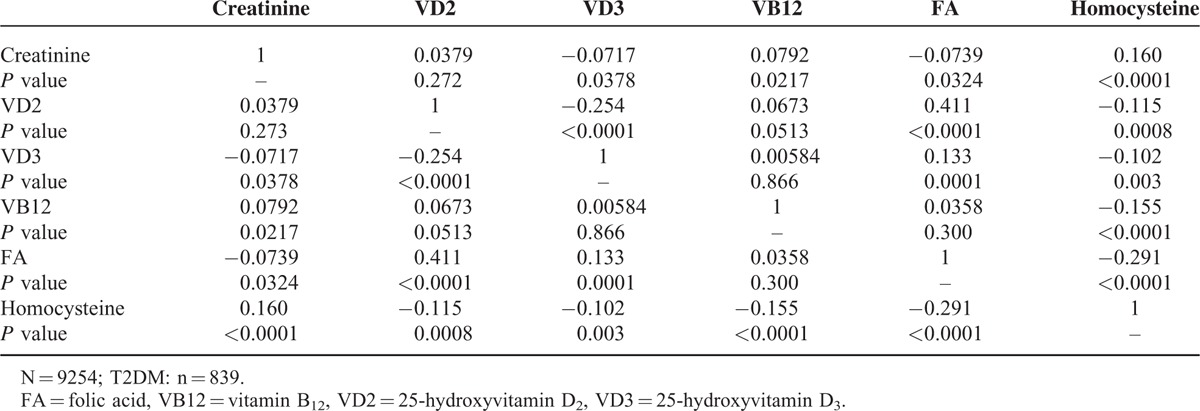
Correlation Coefficients for Comparisons of Serum Biochemical Markers in Patients With Type-2 Diabetes Mellitus

### Correlations Between Serum Biochemical Markers in Hypertension Patients

Correlations between the serum biochemical markers in patients with hypertension are shown in Table 4. Vitamin D2 positively correlated with vitamin B12 and folic acid, and negatively correlated with vitamin D3.Vitamins D3 and B12 positively correlated with folic acid. Homocysteine negatively correlated with folic acid and vitamins D2, D3, and B12. Increases in vitamin D3 and folic acid correlated with decreases in homocysteine and creatinine, which would tend to relieve vascular sclerosis, whereas an increase in vitamin B12 correlated with a decrease in homocysteine only.

**TABLE 4:**
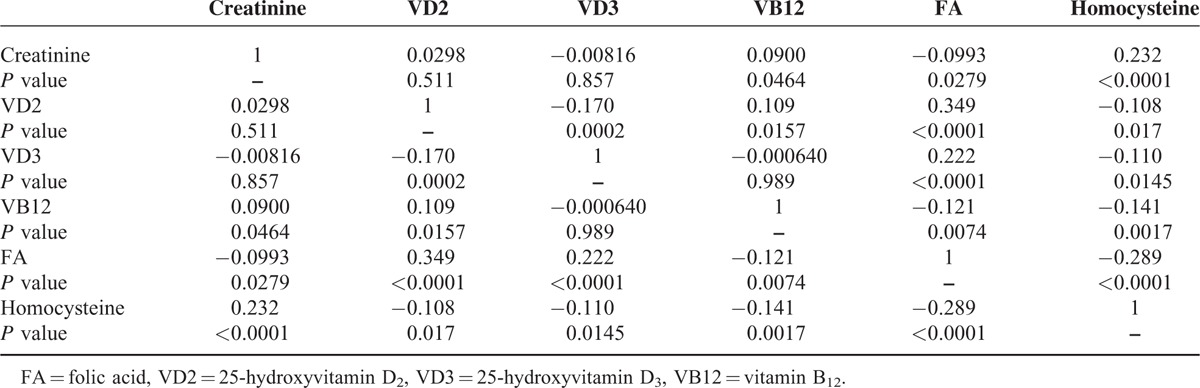
Correlation Coefficients for Comparisons of Serum Biochemical Markers in Patients With Hypertension

### Correlations Between Serum Biochemical Markers in CVD Patients

Correlations between the serum biochemical markers in CVD patients are shown in Table 5. Vitamin D2 positively correlated with vitamin B12 and folic acid, and negatively correlated with vitamin D3. Vitamin D3 positively correlated with folic acid. Vitamin B12 negatively correlated with folic acid. Homocysteine negatively correlated with folic acid and vitamins D2, D3, and B12. An increase in folic acid correlated with decreases in homocysteine and creatinine, which would tend to relieve vascular sclerosis, whereas an increase in vitamin B12 correlated with a decrease in homocysteine only.

**TABLE 5:**
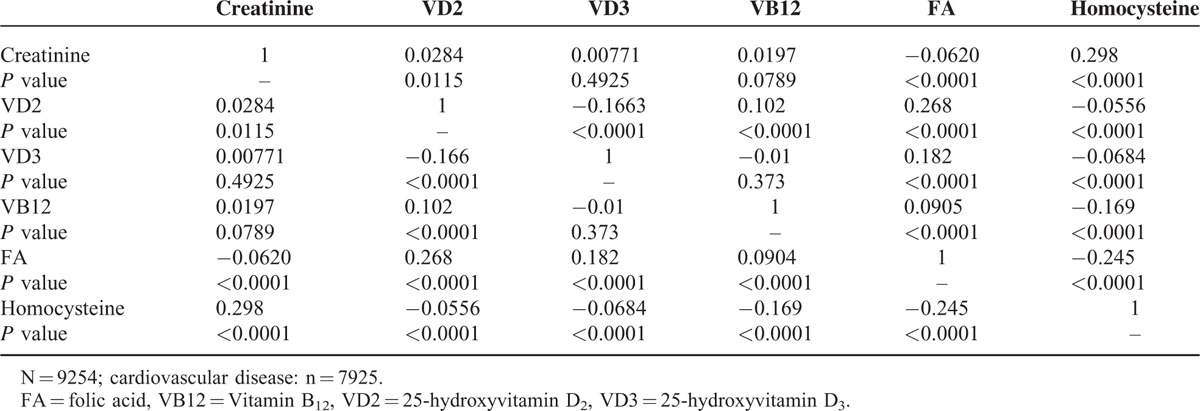
Correlation Coefficients for Comparisons of Serum Biochemical Markers in Patients With Cardiovascular Disease

### Stratified Analysis of the Serum Biochemical Markers Based on the Folic Acid Level in T2DM, CVD, and Hypertension Patients

The results of the stratified analysis based on the folic acid level are shown in Table 6. In T2DM patients, as the level of folic acid increased, the levels of vitamins D2 and D3 increased, and the level of homocysteine decreased. In patients with hypertension, as the level of folic acid increased, the levels of vitamins D2 and D3 increased, and the levels of vitamin B12 and homocysteine decreased. In the CVD patients, as the level of folic acid increased, the levels of vitamins D2, D3, and B12 also increased, and the level of homocysteine decreased.

**TABLE 6:**
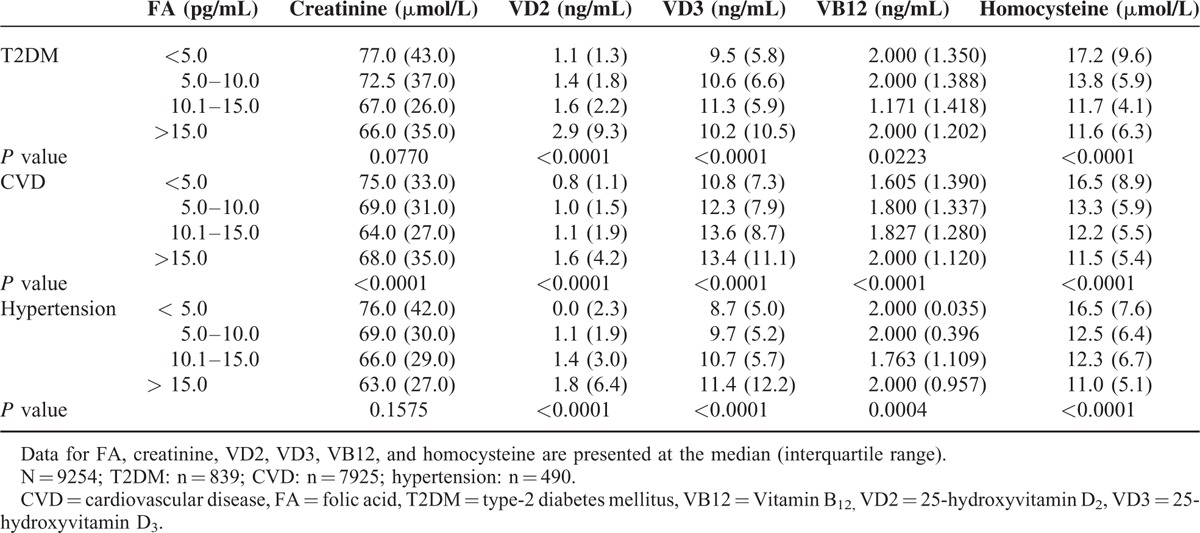
Stratified Analysis of the Serum Biochemical Markers Based on the Level of Folic Acid in Patients With Type-2 Diabetes Mellitus, Hypertension, or Cardiovascular Disease

### Stratified Analysis of the Serum Biochemical Markers Based on Total Vitamin D in T2DM, CVD, and Hypertension Patients

The results of the stratified analysis based on combined levels of vitamins D2 and D3 are shown in Table 7. In the T2DM patients, as the level of total vitamin D increased, the levels of vitamin B12 and folic acid also increased, and the levels of homocysteine and creatinine decreased. In the CVD patients, as the level of vitamin D increased, the levels of vitamin B12, folic acid, and creatinine increased, and the level of homocysteine decreased. In the hypertension patients, as the level of vitamin D increased, the levels of folic acid and creatinine increased, and the level of homocysteine decreased.

**TABLE 7:**
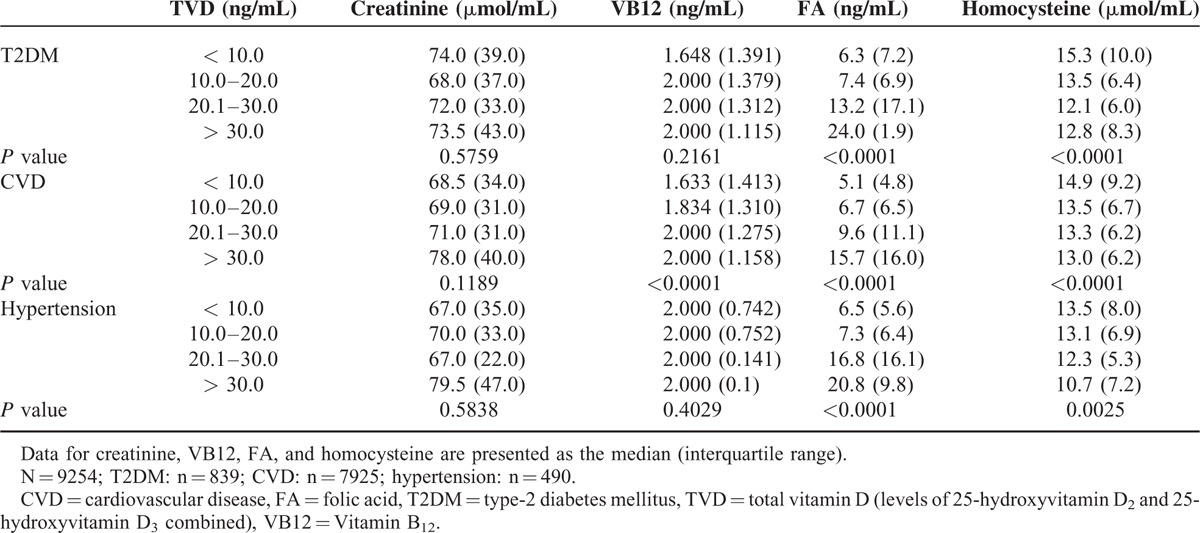
Stratified Analysis of the Serum Biochemical Markers Based on Total Vitamin D in Patients With Type-2 Diabetes Mellitus, Hypertension, or Cardiovascular Disease

## DISCUSSION

In recent decades, the prevalences of T2DM, CVD, and hypertension have increased progressively, making them major public health problems worldwide.^1^ The risk of T2DM is almost 2.5 times higher in persons with hypertension, compared with that in their normotensive counterparts,^24^ and T2DM and hypertension are major contributors to the development of CVD.^1^ The underlying mechanisms by which the levels of vitamins and related serum biomarkers influence the risk of these multifactorial diseases remains largely unclear, and studies of the role of vitamin intake in the development of T2DM, CVD, and hypertension in China are scant. We examined the plasma concentrations of creatinine, homocysteine, B vitamins, and vitamin D in patients with T2DM, hypertension, or CVD in Shanghai, China. Our data suggest that the serum levels of homocysteine, folic acid, and vitamin D are important risk factors for these diseases.

Vitamin D insufficiency is a risk factor for T2DM, CVD, and hypertension.3 Hydroxyvitamin D2 accounts for ∼10% of total vitamin D in serum, a substantial contribution that should be considered in an investigation of disease risk associated with vitamin D. Methods currently used for the detection of vitamins in serum include enzyme-linked immunosorbent assay, radioimmunoassay, and mass spectrometry. Among these, LC-MS/MS methods have demonstrated the highest levels of selectivity and specificity^25^ and have thus become widely used for measuring vitamin D in serum samples. A previous studies have shown that hypovitaminosis D is highly prevalent in China.^22,26,27^ Therefore, we examined the levels of both vitamin D2 and vitamin D3 in our cohorts of T2DM, CVD, and hypertension patients using a highly sensitive LC-MS/MS method to ensure that levels of vitamins D2 and D3 in subjects with vitamin D insufficiencies could be reliably quantified.

In recent years, both clinical studies and studies in animal models have shown that the serum level of vitamin D is associated with T2DM, and observational studies in North America and Europe have shown that vitamin D insufficiency is associated with T2DM^6^ with a relatively high degree of consistency. In our present study, we found that serum levels of vitamin D3 were below the reference range in T2DM patients (Table 1), which is largely consistent with the findings of similar studies performed in Western countries. However, we also found that the serum level of vitamin D2 was elevated in our Chinese T2DM, CVD, and hypertension cohorts, and that a significant negative correlation existed between the serum levels of vitamins D2 and D3 in patients ≥85 years of age. Future studies of the relationship between vitamins D2 and D3 in elderly Chinese patients are warranted to investigate the effect of vitamin D supplementation on the incidence of T2DM in China.

In Chinese patients with hypertension or CVD, we also found that the serum level of vitamin D2 was higher and the level of vitamin D3 lower than the reference range for each (Table 1). Studies in the USA have reported that plasma levels of vitamin D negatively correlated with the risk of developing hypertension or CVD.^8,28,29^ However, these studies did not differentiate between the contributions of vitamin D2 and vitamin D3 to the total serum level of vitamin D in their participants. Therefore, in addition to differences in factors related to their environment, ethnicity, and diet, it is possible that the detection of different species of vitamin D may also have contributed to differences between the results of studies performed in North America and those recorded for our T2DM, CVD, and hypertension cohorts in China.

Homocysteine can directly damage blood vessels and is a significant risk factor for CVD and hypertension.^13–15,30–32^ Homocysteine is an important intermediate in amino acid methylation and transsulfuration pathways that require folic acid and vitamin B12 as key coenzymes. Previous studies have shown that deficiencies in folic acid and vitamin B12 can cause increases in the serum level of homocysteine.^11^ We observed a significant negative correlation between the serum levels of folic acid and vitamin B12 and the serum level of homocysteine in patients ≥50 years of age (Table 2). Therefore, the findings of previous studies in the USA and UK are consistent with our findings in Chinese people. The findings of previous studies regarding the relationship between homocysteine and T2DM have, however, been somewhat inconsistent, with studies reporting elevated,^18,33^ normal,^34–36^ or reduced^37,38^ levels of homocysteine in T2DM patients. We observed an elevated level of homocysteine in our Chinese T2DM patients (Table 1). Future studies of the relationship between homocysteine and T2DM are warranted to clarify the effect of age on the risk of T2DM associated with elevated homocysteine.

We also investigated the relationship between vitamin D and the level of homocysteine in T2DM, CVD, and hypertension patients, which has not been adequately addressed in previous studies. In our stratified analysis, we observed significant differences in homocysteine levels in Chinese T2DM patients based on the total vitamin D level (Table 7). The highest level of homocysteine was observed in the subgroup with the lowest level of total vitamin D (<10 ng/mL),and the lowest level of homocysteine was observed in the subgroup with the highest level of total vitamin D (>30 ng/mL). Furthermore, the inverse was true for levels of folic acid and vitamin B12 in our T2DM patients, and similar trends were also observed in our Chinese CVD and hypertension patients. Future studies are warranted to confirm the inverse relationship between homocysteine and vitamin D in Chinese subjects, and to investigate whether the use of vitamin D supplements might reduce the risks of T2DM, CVD, or hypertension in Chinese people.

Previous studies have shown that folic acid intake inversely correlates with the risk of CVD and stroke.^39,40^ In our stratified analysis-based folic acid level, we observed a negative trend between the level of homocysteine and those of vitamins D2 and D3 in Chinese T2DM, CVD, and hypertension patients, with the serum level of homocysteine decreasing as the levels of vitamins D2 and D3 increased (Table 6). In addition, although no definite trend in the level of vitamin B12 was observed in the T2DM and hypertension patients with regard to an increasing level of folic acid, the serum level of vitamin B12 in CVD patients progressively increased with increases in the serum level of folic acid, suggesting that folic acid and vitamin B12 supplements might be useful for reducing homocysteine levels in Chinese CVD patients or Chinese people at high risk for CVD. However, in the stratified analysis based on age, a significant positive correlation between folic acid and vitamin B12 and significant negative correlations between homocysteine and vitamins D2 and D3 were observed only in patients ≥80 years of age (Table 2). Given that the median ages of the T2DM, CVD, and hypertension groups were 84.4, 83.2, and 83.1 years, respectively, the interpretation of our findings should be limited to elderly Chinese patients only.

In conclusion, the results of our present study revealed specific trends in the correlative relationships between the serum levels of homocysteine, folic acid, and vitamins D2, D3, and B12 in T2DM, CVD, and hypertension patients in Shanghai, China. In all of our patient groups, the level of vitamins D2 was higher, and the level of vitamin D3 was lower the reference range for each, indicating that the contribution of both species should be considered in future investigations of the effects of vitamin D supplements in T2DM, CVD, and hypertension patients. Our linear regression and stratified analyses showed that the highest levels of folic acid and vitamins D2 and D3 correlated with the lowest level of homocysteine in T2DM, CVD, and hypertension patients, whereas the highest level of vitamin B12 correlated with a lowest level of homocysteine in CVD patients only. Our findings warrant future case control studies of the effects of vitamin B and vitamin D supplements in people 50 years of age and older. Our findings also warrant future investigations of the biological mechanism underlying the relationship between vitamin D intake the development of these highly prevalent diseases in elderly patients.
